# The Effect of Regular Intake of Dry-Cured Ham Rich in Bioactive Peptides on Inflammation, Platelet and Monocyte Activation Markers in Humans

**DOI:** 10.3390/nu9040321

**Published:** 2017-03-23

**Authors:** Sara María Martínez-Sánchez, Alfredo Minguela, David Prieto-Merino, María Pilar Zafrilla-Rentero, José Abellán-Alemán, Silvia Montoro-García

**Affiliations:** 1 Cátedra de Riesgo Cardiovascular y Departamento de Nutrición, Facultad de Ciencias de la Salud, UCAM Universidad Católica San Antonio de Murcia, Campus de los Jerónimos, s/n, Guadalupe, 30107 Murcia, Spain; smsanchez@ucam.edu (S.M.M.-S.); mpzafrilla@ucam.edu (M.P.Z.-R.); jabellan@ucam.edu (J.A.-A.); 2 Instituto Murciano de Investigación Biosanitaria Virgen de la Arrixaca (IMIB-Arrixaca), Murcia, Spain; Servicio de Inmunología, Hospital Clínico Universitario Virgen de la Arrixaca, 30120 Murcia, Spain; alfredo.minguela@carm.es; 3 Cátedra de Estadística “Big data”, UCAM Universidad Católica San Antonio de Murcia, 30007 Murcia, Spain; dprieto@ucam.edu

**Keywords:** dry-cured ham, active biopeptides, molecular markers, inflammation, platelet function, monocyte

## Abstract

*Background and aims*: Dietary studies have shown that active biopeptides provide protective health benefits, although the mediating pathways are somewhat uncertain. To throw light on this situation, we studied the effects of consuming Spanish dry-cured ham on platelet function, monocyte activation markers and the inflammatory status of healthy humans with pre-hypertension. *Methods*: Thirty-eight healthy volunteers with systolic blood pressure of >125 mmHg were enrolled in a two-arm crossover randomized controlled trial. Participants received 80 g/day dry-cured pork ham of >11 months proteolysis or 100 g/day cooked ham (control product) for 4 weeks followed by a 2-week washout before “crossing over” to the other treatment for 4 more weeks. Soluble markers and cytokines were analyzed by ELISA. Platelet function was assessed by measuring P-selectin expression and PAC-1 binding after ADP (adenosine diphosphate) stimulation using whole blood flow cytometry. Monocyte markers of the pathological status (adhesion, inflammatory and scavenging receptors) were also measured by flow cytometry in the three monocyte subsets after the interventional period. *Results*: The mean differences between dry-cured ham and cooked ham followed by a time period adjustment for plasmatic P-selectin and interleukin 6 proteins slightly failed (*p* = 0.062 and *p* = 0.049, respectively), notably increased for MCP-1 levels (*p* = 0.023) while VCAM-1 was not affected. Platelet function also decreased after ADP stimulation. The expression of adhesion and scavenging markers (ICAM1R, CXCR4 and TLR4) in the three subsets of monocytes was significantly higher (all *p* < 0.05). *Conclusions*: The regular consumption of biopeptides contained in the dry-cured ham but absent in cooked ham impaired platelet and monocyte activation and the levels of plasmatic P-selectin, MCP-1 and interleukin 6 in healthy subjects. This study strongly suggests the existence of a mechanism that links dietary biopeptides and beneficial health effects.

## 1. Introduction

The food industry sector is well aware of the well-documented positive effects of functional foods or nutraceuticals on diverse physiological functions, apart from their nutritional properties [1]. Improvements in diet and lifestyle to reduce or prevent specific pathological processes such as cardiovascular disease (CVD) have become a goal amongst the general public. Within the last decade, significant improvements in biomarkers related to CV health following the consumption of nutraceuticals—particularly biopeptides—have been described in animal models [2,3,4].

Cardiovascular disease is the leading cause of human mortality and complications worldwide. Hypertension is a well-known clinical risk factor for CVD. Cross-talk between platelets and monocytes is regarded as a crucial pathophysiological mechanism linking thrombosis and inflammation and is believed to mediate, at least in part, the pro-inflammatory action of activated platelets [5]. Experimental data have found that activated platelets enhance cytokine production by monocytes [6] as well as their receptor expression (ICAM-1R and VCAM-1R) and increase their adhesion to the blood vessel wall [7]. However, the importance of monocytes/platelet phenotypical changes in human inflammatory pathophysiology and hypertension remains unclear. Besides, there is limited data on the nutraceuticals effects on inflammatory cells in humans. On the other hand, nutraceuticals such as biopeptides, sterols or polyphenols have been shown to display activities that are beneficial to CV health in humans in terms of blood pressure (BP), lipid metabolism and absorption, oxidative stress, inflammation and haemostasis [8,9,10,11]. The potential health benefits of food-derived bioactive peptides have been extensively reviewed [12,13].

Bioactive peptides are short sequences of 2–20 amino acid long residues that are absorbed through the intestine and distributed through the circulation to produce a local or systemic physiological effect. These peptides are released during food manufacturing, for instance important biochemical changes occur with the curing of meat, including intense proteolysis by muscle peptidases [14,15]. Spanish dry-cured ham particularly, provides a valuable source of active biopeptides that have been tested in vitro and in animal models of hypertension [16,17]. Besides, the results of a number of studies suggest that dry-cured hams may be healthy and could be included as a regular component of the diet [18]. However, any assessment of biological activity has almost exclusively been limited to assays to detect the inhibition of angiotensin I-converting enzyme (ACE) [17] and few human or mechanistic studies have been designed to explore the wider range of activities of such biopeptides in the CV system. Therefore, the current study aims to establish cause–effect relationships between the consumption of dry-cured ham containing bioactive peptides and any physiological effects observed in individuals with pre-hypertension, looking for cellular changes related to the CV pathophysiology other than just clinical parameters. In this study, we focus on changes in monocytes and platelets as they are involved in inflammatory responses and the regulation of the thrombogenic status.

## 2. Material and Methods

### 2.1. Ethics Statement

The clinical study was registered in the Clinical Trials Database (ID: NCT02585089), performed in accordance with the Helsinki Declaration and approved by the Ethics Committee of the Catholic University of Murcia (UCAM, April 2015). All enrolled volunteers provided written informed consent.

### 2.2. Study Design and Subjects

In the context of the 7FP EU Beneficial Effects of Bioactive Compounds in Humans (BACCHUS) project, a two-arm cross-over randomized control trial was assessed at the UCAM, between September 2015 and January 2016. Participants received 80 g/day dry-cured pork ham of >11 months proteolysis (interventional product) and 100 g/day cooked ham (control product) each for 4 weeks. After a two-week washout, the groups exchanged roles for another 4 weeks. The study arms were similar for age, gender, ethnicity, body mass index and BP, which allowed comparisons to be made between them. Thirty-eight apparently healthy Caucasian men and women from the University staff, aged 40–55 years, in good general health and with pre-hypertension were recruited (systolic and diastolic BPs above 125 and 80 mmHg, respectively).

Exclusion criteria were smokers, *Diabetes mellitus*, diagnosed and treated hypertension, history of cardiovascular events (stroke, myocardial infarction or peripheral vascular disease), cancer and inflammatory diseases. Volunteers whose medications included anti-hypertensives, antiaggregants, anticoagulants, antidepressants, anti-cholinergic or anti-spasmodic agents, the regular use of medication affecting intestinal motility, vasodilators, lipid lowering therapies or fish oil supplements (all other supplements were assessed on a case by case basis) were also excluded.

A list of restricted foods was given at the beginning of the study in order to avoid the excessive consumption of salt and other cured meat products. Volunteers were asked not to change their dietary patterns and lifestyle during the study. The individuals did not know about the purpose of the study or which of the hams was supposed to be the interventional product. The volunteers were enrolled by simple randomization by only one investigator and the randomization sequence was concealed until the end of the statistical analysis.

### 2.3. Meat Products

Spanish dry-cured ham with a controlled salt content after >11 months dry-curing process/proteolysis was the interventional product and cooked ham was the control product.

The Spanish dry-cured ham after an >11 month dry-curing process contained 25% less salt than similar products on the market (4.38% vs. approx. 5.5%). As regard the cooked ham, it contained 2.61% salt, which was lower than the 3.6 g salt per 80 g intake of dry-cured ham. In order to counteract the humidity and salt, a higher amount of cooked ham (control) was given (100 g/day). Hams were manufactured and supplied by the local meat Company *ElPozo Alimentación* (Alhama de Murcia, Spain) especially for the present clinical study in daily vacuum bags and labelled with safety information (best by date, nutritional composition and storage information). The presence/absence of active biopeptides in the meat products were tested and sequenced using one-dimensional reversed-phase liquid chromatography combined with nano-liquid chromatography and an electrospray ionization source coupled to a quadrupole time-of-flight mass spectrometer at the *Instituto de Agroquímica y Tecnología de Alimentos* (CSIC, Valencia) [19] (Table 1).

### 2.4. Blood Sampling and Biochemical Determinations

For laboratory analysis, 8 h fasting peripheral venous blood samples were collected at four time-points from all participants before and after each period (interventional/control product). Venous blood was collected in EDTA and citrate (0.15%) Vacutainer^®^ tubes. Blood was centrifuged at 3500 rpm for 15 min at room temperature to obtain platelet-poor plasma. Plasma aliquots were stored at −70 °C to allow ELISA batch analysis.

### 2.5. Flow Cytometry

(a) In vitro assessment of platelet activation:

Freshly collected citrated blood (450 μL) was incubated with 0.02 mmol/L ADP (adenosine diphosphate) for 2 min. Surface expression of membrane constitutive CD42a (GP IX) and platelet activation markers (P-selectin and PAC-1 binding) were then assessed as already published [20]. After incubation, blood (5 μL) was labelled with 0.05 μg anti-CD42a-PerCP (clone Beb1), anti-CD62P-APC (clone AK-4), PAC-1-FITC (clone PAC-1) (which recognizes the conformationally activated GPIIβ/IIIα) (all from BD Biosciences, Oxford, UK) for 15 min, diluted with 1 mL filtered PBS and then analyzed in a FACS Canto II flow cytometer (Becton Dickinson, Oxford, UK). Twenty thousand platelets were counted in each sample run.

**Figure 1:**
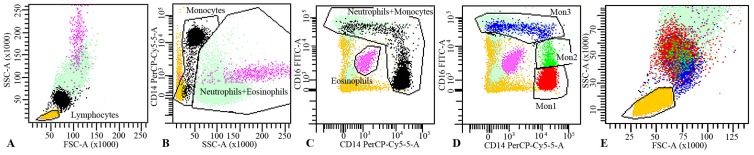
Gating strategy to identify the three peripheral blood monocyte subsets (Mon1, Mon2 and Mon3). (**A**) FSC/SSC-dotplot was set to identify leukocytes and select lymphocytes; (**B**) Leukocytes were plotted in a SSC/CD14-dotplot to select high-SSC cells as Neutrophils+Eosinophils and intermedia-SSC and CD14++/+/− as Monocytes; (**C**) Leukocytes were also plotted in a CD14/CD16-dotplot to select Eosinophils (auto-florescent cells) and Monocyte+Neutrophils, clearly separated from the rest of the blood cell subsets. Logical combination of these gates allowed discrimination between Lymphocytes, Monocytes, Neutrophils and Esosinophils; (**D**) To identify the three monocyte subsets, a CD14/CD16 dotplot was used: Mon1 (CD14++CD16−, red), Mon2 (CD14++CD16+, green) and Mon3 (CD14+CD16++, blue); (**E**) Zoom area of mononuclear cells showing the three monocytes subsets clearly differentiated from lymphocytes and neutrophils.

(b) In vitro assessment of monocyte activation:

Mouse anti-human monoclonal fluorochrome-conjugated antibodies against CD16-Alexa Fluor 488 (clone DJ130c, AbDSerotec, Oxford, UK), CD14-PerCP (clone MфP9, BD Biosciences, Oxford, UK) were mixed in different tubes with 100 μL freshly collected EDTA blood. The above antibodies were mixed with PE-conjugated antibodies against Toll-like receptor-4 (TLR4), intercellular cell adhesion molecule-1 receptor (ICAM-1R) and CXCR4 (all from R&D Systems); APC-conjugated antibodies against interleukin-6 receptor (IL6R), vascular cell adhesion molecule-1 receptor (VCAM-1R) and CD163 (all from R&D Systems). After incubation for 15 min, red blood cells were lysed by 2 mL of lysing solution^®^ (BD Biosciences) for 15 min, diluted with PBS and immediately analyzed by flow cytometry. Expression of the surface markers was quantified as mean fluorescence intensity (MFI) on different monocyte subsets (CD14++CD16− (Mon1), CD14++CD16+ (Mon2), and CD14+CD16++ (Mon3)). The gating strategy excluded neutrophils, eosinophils, lymphocytes and other granulocytes from the analysis (Figure 1). The method was assessed in a BD FACS Canto II flow cytometer (Becton Dickinson, Oxford, UK) as previously described [21], acquiring 120.000 monocytes per sample. 

### 2.6. Plasma Markers

Plasma samples (citrated and EDTA) were used to quantify the levels of soluble platelet and monocyte adhesion markers (P-selectin and VCAM-1, respectively), high sensitive interleukin-6 (hs-IL-6) and monocyte chemoattractant protein 1 (MCP-1) by enzyme-linked immunosorbent assay (ELISA).

All measurements were performed using recognized high quality human Affymetrix kits (eBioscience, Carlsbad, CA 92008, USA): VCAM-1 antigen (Cat. No. BMS232), hs-IL6 antigen (Cat. No. BMS213HS), P-selectin antigen (Cat. No. BMS219/4) and MCP-1 antigen (Cat. No. 88739922), according to the manufacturer’s recommendations. The lower limits of detection of hs-IL-6, P-selectin, VCAM-1 and MCP-1 were 0.03 pg/mL, 0.2 ng/mL, 0.6 ng/mL and 7 pg/mL, respectively.

### 2.7. Statistical Analyses

Categorical variables are expressed as frequency (percentage) of volunteers. Continuous markers are expressed as mean ± standard deviation (SD). Extremely skewed variables were Log transformed. For each continuous marker, a regression of values after meat product consumption adjusted for values before consumption was performed. By centering both variables on the mean before consumption, the intercept of the model estimates the average change after consumption. The relative mean change (mean change/mean before –1) was calculated in order to plot variables with different scales on the same graph.

To estimate the differences of average changes between control and intervention groups, a binary variable (control = 0, intervention = 1) was included in the model. Another binary variable was included to adjust for the potential period effect. Furthermore, a random effect term by individual was used to account for the repeated measures. We checked the model assumptions on the residuals with plots and normality tests. R 3.3.1 software was used for the statistical analyses (R Foundation for Statistical Computing, Vienna, Austria).

## 3. Results

Clinical characteristics, including demography and BP, of the study population, are shown in Table 2. Thirty-eight subjects (44.3 ± 5.3 years old; 82% males) were included in the study. Readings of systolic BP were above 125 mmHg, considered as normal-high blood pressure or pre-hypertension.

The baseline parameters of the participants were compared to data obtained after 4 weeks of consuming dry-cured pork ham and cooked ham (Table 3). Body mass index and fat content were not affected by the interventional product nor by the control product (*p* > 0.05, data not shown).

### 3.1. Plasmatic Markers

**Figure 2:**
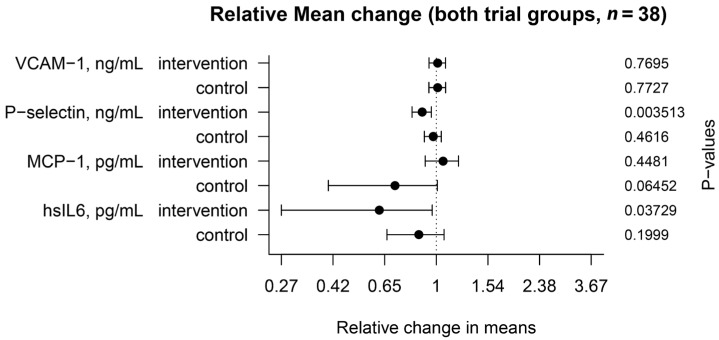
Mean change relative to baseline time in soluble plasmatic marker levels in the interventional and control groups. The values were compared after participants had ingested 80 g/day of dry-cured ham or 100 g/day of cooked ham (*n* = 38) for 4 weeks (+95% confidence interval).

A comparison between plasma biochemical markers before and after dry-cured ham intake is shown in Table 3 and Figure 2. After 4 weeks of regular dry-cured ham intake and adjusting for a potential period effect, soluble P-selectin (sCD62P) concentration was markedly lower than in baseline conditions (*p* = 0.0035). The mean difference of soluble P-selectin levels was still slightly decreased between interventional and control periods (*p* = 0.0625). Besides, MCP-1 levels were found unaltered after the interventional period, but when the cooked ham intake was included, a significant increase in MCP-1 levels was observed (*p* = 0.0234). Decreased concentrations of the soluble inflammatory mediator (IL6) were measured in plasma after the interventional period (*p* = 0.037). Such an effect was still shown after adjusting with the control cooked ham period (*p* = 0.049). In contrast, there were no significant differences in plasma concentrations of soluble VCAM-1 after dry-cured ham nor cooked ham intake.

### 3.2. Effect on Platelet Activation

**Figure 3:**
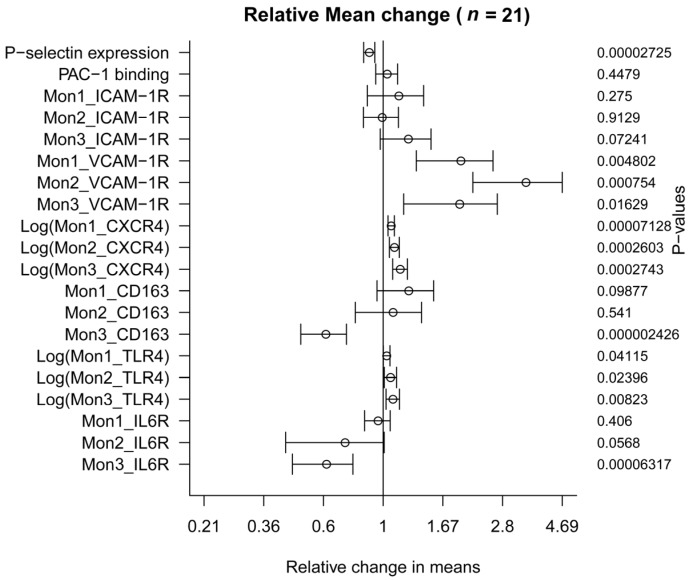
Mean change relative to baseline time in platelet and monocyte activation markers. The values were compared after participants had ingested 80 g/day of dry-cured ham (*n* = 21) for 4 weeks (+95% confidence interval).

The regular consumption of bioactive peptides resulted in a significant decrease in P-selectin (CD62P) expression by 11.17% (55.24 points) in platelets stimulated with ADP (*p* = 0.0000272) (Table 4 and Figure 3). However, changes in the glycoprotein IIb/IIIα expression (PAC-1 monoclonal antibody binding) did not change after the 30 days’ intervention (*p* = 0.447).

### 3.3. Effect on Monocytes Markers

There was no difference in the expression of surface ICAM-1 receptor between the two time points in the different subtypes of monocytes. Similar data were also obtained for CD163 expression (Table 4). However, expression of CD163 in Mon3 decreased significantly by 39% (*p* = 0.0000024) (Figure 3).

Besides, the expression levels of VCAM-1 receptor and CXCR4 were significantly higher for all three subsets compared to baseline conditions (all *p* < 0.02). Dry-cured ham intake produced an important increase of 343% in VCAM-1R levels in Mon 2 (*p* = 0.00075) (Figure 3). Expression levels of TLR4 in the three monocytes subsets were also strongly affected after the intervention (all *p* < 0.05) (Figure 3). Similarly, IL6R expression was down-regulated by 39% in Mon3 (*p* = 0.0000632) but these differences did not reach statistical significance for the other two monocyte subsets, Mon1 and Mon2 (Table 4), although a trend was found for Mon2 (*p* = 0.0568).

## 4. Discussion

Dietary bioactives are compounds of food origin that safely deliver health benefits, although their mechanisms of action are usually poorly understood. Generally, meat and meat products are not ordinarily associated with health benefits [22]. However, in vitro tested health-promoting bioactive peptides are naturally generated from meat proteins during curing or fermentation. Moreover, it is a challenge for the food industry to identify the functionality of bioactives, in order to make products healthier and, possibly to claim that they may be regarded as functional foods. Therefore, the aim of this investigation was to evaluate the effect of the regular consumption of dry-cured pork ham containing characterized active biopeptides among other possible bioactives on platelet activation, monocytes phenotype and plasma markers of inflammation and activation.

The presence of naturally active biopeptides in the meat product was confirmed by a peptidomic strategy using mass spectrometry techniques [19]. Cooked ham was chosen as the control product because active biopeptides are not released during its manufacture, as confirmed here. To our knowledge, no previous study has associated the regular consumption of meat peptides with in vivo cellular changes, even though several in vitro studies suggested possible action mechanisms [17,23,24].

Impaired platelet function results in a higher degree of degranulation and translocation of P-selectin (CD62P) to the outer plasmatic membrane, both of which have been related to CV pathological conditions such as hypertension, diabetes, myocardial infarction, heart failure and stroke [25]. Accordingly, P-selectin has also been proposed as a novel therapeutic target in vascular disease [26]. One of the more significant findings to emerge from this study is that the intake of dry-cured ham enriched in active biopeptides is related to a decrease in the platelet expression of this activation marker, P-selectin. Moreover, the findings also point to a reduction of soluble P-selectin after the interventional period, even adjusting with the cooked ham intake. This data also supports a role for active biopeptides in modulating in vivo platelet function. The ability to inhibit platelet function has previously been confirmed ex vivo in the presence of other vegetable bioactives [27]. Nonetheless, it is unclear why this occurs or what role this response might play in pathological conditions, other than in healthy individuals with normal-high non-treated hypertension. Thereby, the current study opens up an interesting avenue for further research into the mechanism(s) of these dietary peptides in platelet phenotype and function.

The roles of monocytes in CVD are diverse, including their involvement in inflammatory responses and the regulation of the thrombogenic status. In experimental hypertension, monocytes increase their adhesion to the endothelium integrins (ICAM-1, VCAM-1) through their receptors (ICAM-1R and VCAM-1R). Moreover, monocytes have also been implicated in physiologically beneficial processes related to the scavenging of pathological material, angiogenesis and repair through stromal derived factor-1 (SDF1) binding to CXCR4. The diversity of monocytes functions can be partly attributed to the existence of different monocytes subsets (Mon1, Mon2 and Mon3) that can be distinguished according to specific phenotypic and functional properties [28]. In this line, the second major finding of the current study was that the consumption of dry-cured ham impaired the basal expression of several markers in circulating monocytes. For instance, the expression of the integrin α4 receptor (VCAM-1R) and CXCR4 (SDF1 receptor) increased significantly in the three subtypes of monocytes. Recent investigations found that VCAM-1R and CXCR4 in monocytes were higher in healthy volunteers compared to individuals with coronary artery disease (CAD) [21], confirming an improvement of the physiological status after the long-term consumption of dry-cured ham. CD163 and TLR4 are proteins involved in immunity, scavenging and the recognition of antigens [29]. The TLR4 expression also increased after the intervention in all the three monocyte subsets. The expression of CD163 was higher after the intervention in the minority Mon3. Concerning the expression of IL6R, levels remained unaltered after the intervention in the main subset Mon1 but it significantly decreased in Mon3 and a trend was shown for Mon2, once more in agreement with previous results with healthy volunteers towards CAD patients [21]. Whether IL6R levels decrease as a consequence of lower transcription cannot be formally demonstrated here. Nonetheless, greater IL6 binding to IL6R might not be the case because the plasmatic levels of IL6 were substantially lower after the regular consumption of cured meat adjusted with a potential period effect. Neither can the effects be attributed to observer bias or lifestyle confounders. In relation to that, the current analysis also identified increased plasmatic MCP-1 levels, which regulate the migration of monocytes, between the intervention and control groups.

Taken together, those observations seem to suggest intake-dependent changes in monocytes and an improvement in the inflammatory status.

### Limitations

Differences in the molecular parameters were only shown after the interventional period of time. However, plasmatic markers were analyzed after the intake of both meat products, allowing a valid estimate of the dry-cured ham intake effect. Although irrelevant from the clinical point of view, it is important to mention that volunteers took lower salt amounts during cooked ham intake (1 g/day less), compared to the interventional period. The effect of dry-cured ham consumption on platelet and monocytes phenotype cannot be directly attributed to active biopeptides; nonetheless, it is important to underline the broad range of sequenced peptides contained in the dry-cured ham and absent in the control product. The ideal quantity of biopeptides from any origin that would provide beneficial effects in humans has not been determined; neither has the amount of biopeptides contained in the ham. Nevertheless, the study was designed to involve the highest feasible daily intake of dry-cured meat.

## 5. Conclusions

As the era of molecular nutrition unfolds, a greater understanding of how these foods and their components influence cellular mechanisms will surely arise. Current molecular approaches, such as flow cytometry, can aid the scientific community in gaining a deeper understanding related to the effects of active compounds over a variety of cell types, tissues and pathological conditions. Such information will be critical in the development of healthier products for reducing the CV burden. In particular, the present results lead to the conclusion that active biopeptides have potential therapeutic effects in the vascular system, and help identify future molecular targets for the prevention of CV risk factors in healthy individuals. In addition, whether these new categories of active compounds affect platelet function, monocytes phenotype and inflammation requires further well-controlled interventional studies. It is therefore envisaged that future in vitro experiments should try to confirm the association of these factors and that in vivo studies will be carried out in patients with high CV risk.

## Figures and Tables

**Table 1:** Analysis of Active Biopeptides in the Meat Products.

Active Biopeptides	Dry-Cured Ham	Cooked Ham
KAAAAP	Identified	N.D.
AAPLAP	Identified	N.D.
KPVAAP	Identified	N.D.
VPPAK	Identified	N.D.
KPGRP	Identified	N.D.
IAGRP	N.D.	N.D.
PAAPPK	Identified	N.D.
TGLKP	N.D.	N.D.
AAATP	Identified	N.D.
KAAAATP	N.D.	N.D.

N.D.: not detected.

**Table 2:** Clinical Characteristics of Healthy Volunteers with Normal-High Hypertension.

*n* = 38	Volunteers
Age	44.3 ± 5.3
Gender (male)	82%
BMI, (kg/m^2^)	27.02 ± 3.9
Fat content, %	25.08 ± 7.3
Systolic BP, mmHg	137.4 ± 10.6
Diastolic BP, mmHg	80.0 ± 5.8

BMI: Body Mass Index; BP: Blood Pressure.

**Table 3:** Plasmatic Parameters with Volunteers in Both Trial Arms (Mean Difference After (1) Dry-Cured Ham Intake, (2) Cooked Ham and (1–2) Difference).

*n* = 38	Period of Intervention	Mean Difference	Confidence Interval		*p* Value
			Low 95%	Upp 95%	
*Plasmatic markers, ELISA*					
P-selectin, ng/mL	(1) Dry-cured	−5.00	−8.19	−1.80	0.003513
	(2) Control	−1.31	−4.60	2.10	0.461629
	(1)–(2)	−3.69	−7.36	0.02	0.062522
VCAM-1, ng/mL	(1) Dry-cured	3.23	−17.97	24.42	0.769504
	(2) Control	3.41	−19.33	26.15	0.772709
	(1)–(2)	−0.19	−25.61	25.24	0.988745
MCP-1, pg/mL	(1) Dry-cured	5.45	−8.35	19.25	0.448144
	(2) Control	−14.39	−29.20	0.42	0.064524
	(1)–(2)	19.84	3.28	36.40	0.023489
hs-IL6, pg/mL	(1) Dry-cured	−0.60	−1.15	−0.05	0.037294
	(2) Control	−0.39	−0.96	0.19	0.199947
	(1)–(2)	−0.52	−0.77	−0.04	0.049448

BMI: Body Mass Index; VCAM-1: Vascular Cell Adhesion Molecule 1; MCP-1: Monocyte Chemoattractant Protein-1; hs-IL6: high-sensitivity Interleukin 6.

**Table 4:** Cellular Parameters of the Volunteers (Baseline and Mean Difference after Consuming Dry-Cured Ham for 4 Weeks).

*n* = 21	Baseline (before Dry-Cured Ham)	Mean Difference	Confidence Interval		*p* Value
			Low 95%	Upp 95%	
*Platelet activation markers*					
P-selectin expression, MFI	492.67	−55.24	−76.32	−34.16	0.0000272
PAC-1 binding, MFI	426.12	15.18	−26.33	56.68	0.447
*Monocyte activation markers (n = 21)*					
Mon1_ICAM-1R, MFI	3799.22	552.94	−484.15	1590.03	0.275
Mon2_ICAM-1R, MFI	6325.33	−49.33	−989.93	891.26	0.912
Mon3_ICAM-1R, MFI	4579.78	1114.66	−113.87	2343.20	0.072
Mon1_VCAM-1R, MFI	672.70	643.25	222.89	1063.61	0.00480
Mon2_VCAM-1R, MFI	1243.75	3022.30	1453.88	4590.72	0.00075
Mon3_VCAM-1R, MFI	2048.55	1917.80	397.35	3438.25	0.01629
Mon1_CXCR4, MFI	1152.70	1019.90	545.43	1494.38	0.0000713 *
Mon2_CXCR4, MFI	2415.00	4244.17	1313.45	7174.89	0.0002603 *
Mon3_CXCR4, MFI	2215.55	9601.76	1498.90	17,704.61	0.0002743 *
Mon1_CD163, MFI	1376.20	340.70	−70.88	752.28	0.098
Mon2_CD163, MFI	1838.90	164.94	−392.83	722.70	0.540
Mon3_CD163, MFI	2606.90	−1016.48	−1325.88	−707.08	0.0000024
Mon1_TLR4, MFI	1050.57	261.05	−44.91	567.00	0.0411524 *
Mon2_TLR4, MFI	1915.72	2061.10	52.87	4069.33	0.0239622 *
Mon3_TLR4, MFI	1611.00	3266.19	468.86	6063.52	0.0082297 *
Mon1_IL6R, MFI	576.45	−24.60	−85.34	36.14	0.406
Mon2_IL6R, MFI	1186.40	−332.05	−674.78	10.68	0.0568
Mon3_IL6R, MFI	3820.70	−1475.70	−2074.37	−877.03	0.0000632

ICAM-1R: Intercellular Adhesion Molecule-1 Receptor; VCAM-1R: Vascular Cell Adhesion Molecule-1 Receptor; TLR4: Toll-Like Receptor 4; IL6R: Interleukin 6 Receptor; MFI: Mean Fluorescence Intensity. * Data were log transformed before analysis.

## Abbreviations

ACE	Angiotensin I Converting Enzyme
ADP	Adenosine diphosphate
BMI	Body Mass Index
BP	Blood Pressure
CAD	Coronary artery Disease
CVD	Cardiovascular Disease
FSC/SSC	Forward-scattered light/Side-scattered light
hsIL6	high-sensitivity Interleukin 6
ICAM-1	Intercellular Adhesion Molecule-1
MCP-1	Monocyte Chemoattractant Protein-1
MIF	Mean Fluorescence Intensity
Mon	Monocyte
SDF1	Stromal Derived Factor-1
TLR4	Toll-Like Receptor 4
VCAM-1	Vascular Cell Adhesion Molecule 1
UCAM	Catholic University of Murcia
